# Computational disease modeling – fact or fiction?

**DOI:** 10.1186/1752-0509-3-56

**Published:** 2009-06-04

**Authors:** Jesper N Tegnér, Albert Compte, Charles Auffray, Gary An, Gunnar Cedersund, Gilles Clermont, Boris Gutkin, Zoltán N Oltvai, Klaas Enno Stephan, Randy Thomas, Pablo Villoslada

**Affiliations:** 1 Computational Medicine group, Department of Medicine, Center for Molecular Medicine, Karolinska University Hospital, Solna, Stockholm, Sweden; 2 Institut d'Investigacions Biomèdiques August Pi I Sunyer (IDIBAPS), Villarroel 170, 08036 Barcelona, Spain; 3 Functional Genomics and Systems Biology for Health, LGN-UMR 7091, CNRS and Pierre & Marie Curie University of Paris VI, 7, rue Guy Moquet – BP8 – 94801 Villejuif cedex, France; 4 Division of Trauma/Critical Care, Department of Surgery, Northwestern University Feinberg School of Medicine, Chicago, IL, USA; 5 Department of clinical and experimental medicine, Cell biology and diabetes research centre, Linköping University, Linköping, SE58185, Sweden; 6 Center for Inflammation and Regenerative Modeling and CRISMA laboratory, Department of Critical Care Medicine, University of Pittsburgh, 3550 Terrace, Pittsburgh, PA 15261, USA; 7 Group for Neural Theory, DEC-ENS, 3 rue d'Ulm, 75005 Paris, France; 8 Departments of Pathology and Computational Biology, University of Pittsburgh, 3550 Terrace St., Pittsburgh, PA 15261, USA; 9 Laboratory for Social and Neural Systems Research, Institute for Empirical Research in Economics, University of Zurich, Zurich, Switzerland; 10 Wellcome Trust Centre for Neuroimaging, Institute of Neurology, University College London, London, UK; 11 CNRS FRE 3190 IBISC, Evry, France; and University Evry-Val d'Essonne, Evry, France; 12 Institut d'Investigacions Biomèdiques August Pi I Sunyer (IDIBAPS), Hospital Clinic. Villarroel 170, 08036 Barcelona, Spain

## Abstract

**Background:**

Biomedical research is changing due to the rapid accumulation of experimental data at an unprecedented scale, revealing increasing degrees of complexity of biological processes. Life Sciences are facing a transition from a descriptive to a mechanistic approach that reveals principles of cells, cellular networks, organs, and their interactions across several spatial and temporal scales. There are two conceptual traditions in biological computational-modeling. The bottom-up approach emphasizes complex intracellular molecular models and is well represented within the systems biology community. On the other hand, the physics-inspired top-down modeling strategy identifies and selects features of (presumably) essential relevance to the phenomena of interest and combines available data in models of modest complexity.

**Results:**

The workshop, "ESF Exploratory Workshop on Computational disease Modeling", examined the challenges that computational modeling faces in contributing to the understanding and treatment of complex multi-factorial diseases. Participants at the meeting agreed on two general conclusions. First, we identified the critical importance of developing analytical tools for dealing with model and parameter uncertainty. Second, the development of predictive hierarchical models spanning several scales beyond intracellular molecular networks was identified as a major objective. This contrasts with the current focus within the systems biology community on complex molecular modeling.

**Conclusion:**

During the workshop it became obvious that diverse scientific modeling cultures (from computational neuroscience, theory, data-driven machine-learning approaches, agent-based modeling, network modeling and stochastic-molecular simulations) would benefit from intense cross-talk on shared theoretical issues in order to make progress on clinically relevant problems.

## Background

The recent "ESF Exploratory Workshop on Computational disease Modeling" [1] workshop in Barcelona (Sept. 24–26, 2008) brought together modelers, experimentalists and clinicians to discuss how multi-factorial human diseases (including multiple sclerosis, cancer, cardiovascular and kidney diseases, diabetes, sepsis, allergy, schizophrenia and addiction) can be modeled given the currently available knowledge and data. Experts covered areas such as molecular network modeling, computational neuroscience, pharmacokinetic and pharmacodynamic modeling, hierarchical modeling and agent-based modeling.

Successful modeling of diseases is greatly facilitated by standards for data-collection and storage, interoperable representation, and computational tools enabling pattern/network analysis and modeling. There are several important initiatives in this direction, such as the ELIXIR program [2] providing sustainable bioinformatics infrastructure for biomedical data in Europe. Similar initiatives are in progress in the USA and Asia. Yet these efforts in themselves are not sufficient, as the predictive understanding of complex diseases requires computational modeling and representation of these data. However, despite ongoing efforts, there are deep and unsolved conceptual and theoretical issues regarding the use of computational modeling and representation of data to advance the predictive understanding of complex diseases. We uncovered a few core problems that have not been sufficiently recognized, which must be addressed when trying to leverage the available and growing amounts of relevant biological information.

### Model selection and parameter uncertainty

Across different application areas, a key question concerns the handling of model uncertainty. This refers to the fact that for any biological system there are numerous competing models. Any discursive model of a biological system therefore involves uncertainty and incompleteness. Computational model selection has to cope systematically with the fact that there could be additional relevant interactions and components beyond those that are represented in the discursive model. For instance, there is often insufficient experimental determination of kinetic values for mechanisms contemplated in a verbal model, leading to serious indetermination of parameters in a computational model. Hence, biological models, unlike models describing physical laws, are as a rule highly over-parameterized with respect to the available data. This means that different regions of the parameter space can describe the available data equally well from a statistical point-of-view. Because of these interdependencies, interpreting parameter estimates of individual models can be very difficult. There are good reasons to believe that such interdependencies are unavoidable (and to some degree even desirable, to increase robustness against lesions) in biological systems [3].

A successful strategy in computational neuroscience has been to identify minimal models that adequately describe and predict the biology, but at the potential price of selecting a too narrowly focused model. This approach is justified if adequate knowledge of the underlying mechanisms involved in a given condition exists. In situations where the biology is less well-characterized one must consider and compare several plausible model structures. An alternative approach, recently employed within the systems biology and computational neuroscience fields, is to search for parameter dimensions (as opposed to individual parameter sets) that are important for model performance. This concept of *model ensembles *represents a promising approach. The process of characterizing parameter values is applied to each model structure and the resulting ensemble is the collection of model structures and their associated probabilistic parameter distributions. Stochastic search of parameter space using a variety of techniques (e.g. Markov chain Monte Carlo-based) seems to be state of the art. Multi-start convex optimizations or particle swarm optimization (PSO) algorithms locate a potentially large number of local minima of a user-defined, biologically relevant objective functions. However, they do not offer assurance of adequate coverage of parameter space, nor do they have the asymptotic property of resulting in a probability density function in parameter space. It is not yet clear under what conditions an optimization is most useful. Furthermore, there are no clear ways to combine selected models to create a consensus ensemble used to formulate predictions: choosing the "best" structure or weighing competing structures based on their relative fitness. Model-guided experimental design appears a promising avenue for clarifying model structure.

Model selection is therefore important to prevent over-fitting and to distinguish between competing explanations. Bayesian model selection is utilized in computational neuroimaging [4] and may also prove useful in systems biology. There is also a bias towards mechanistic and molecular models in systems biology. Models should not only be mechanistic, but also allow for experimental validation of the mechanisms they propose. This means that their components should be at a level of description that allows for the design and/or inclusion of experimental perturbations using current experimental techniques. More generally, a mechanistic model is not very helpful unless there are experimental means to assess its predictive validity (over and beyond its face validity and construct validity; these different types of validity are not always distinguished, although the distinction is very important).

### Hierarchical models

The second major theme concerned the development of hierarchical models, spanning several scales of biological organization from intracellular molecular networks and cell-to-cell interactions to interacting tissues and organs of the whole body.

Much attention was devoted to organ-level models or diseases (e.g., multiple sclerosis, allergic rhinitis and sepsis), and on the Virtual Physiological Human Project in particular. Methodologies, like co-simulation which allows for parallel simulation at different time-scales in different modules, and modeling environments for integrative models including different types of equations (ODEs, PDEs, SDEs etc) were discussed and evaluated. It appears that the systems biology community focuses on intracellular networks whereas computational neuroscience emphasizes top-down modeling. Presentations on schizophrenia and nicotine addiction used very simple, top-down models to explain complex phenomena and offered useful predictions. By characterizing systems properties, behavior constraints can propagate to lower scales and may reduce the number of solutions consistent with experimental observations. This potential gain has not been sufficiently exploited when trying to model large-scale high-throughput data. It must also be recognized that top-down models of insufficient richness may excessively constrain model space and lose predictive ability.

There is a lack of theory for how to integrate model selection with constraint propagation across several layers of biological organization. Development of such a theory could be useful in modeling complex diseases even when only sparse data is available. One useful practical first approximation is the notion of disease networks – i.e. network representations of shared attributes among different diseases and their (potential) molecular underpinnings. This approach may provide both bottom-up and top-down constraints for understanding complex diseases, enabling a question-driven middle-out approach advocated by Sydney Brenner, Denis Noble and others. There are multiple well-known examples for these, including obesity-diabetes, Gaucher disease-Parkinson disease, etc. At the same time, alteration of the physiome by a given disease can also lower the chance of developing another disease, e.g. sickle cell disease. (Hgb S) and malaria infection. Agent-based approaches also offer the possibility of multi-scale synthesis by providing a modular framework for dynamic knowledge representation.

### Scientific cultures and the future

During the discussions at the workshop, striking differences in the scientific "culture" of sub-disciplines of theoretical biology were observed. This difference was most noticeable with regard to different model building approaches in computational systems biology and computational neuroscience. In the former, much attention is given to formal methods of model selection and data-driven model construction. In contrast, in computational neuroscience (with the notable exception of computational neuroimaging), formal model selection methods are almost completely absent. The historical roots of such differences between the two sub-disciplines have recently been reviewed [5].

In summary, high priorities for the future of complex disease modeling are to make progress on model selection and hierarchical modeling. There is a need for a forum (ESF network and/or intercontinental efforts) where control theory, physics and applied mathematics can stimulate method development across different areas of computational biology. Advances in biosystems theory can nurture current modeling efforts and therefore be more clinically useful. The study of complex diseases challenges researchers with unexpected findings and questions not previously envisioned when studying only basic biological process. Towards that end, we believe that studying groups of patients with common patterns of disease is a useful concept and is a more tractable target in the short term than truly understanding individualized dynamics.

## Authors' contributions

All authors JT, AC, CA, GA, GC, GC, BG, ZO, KES, RT, PV provided input with the key ideas in the manuscript. JT wrote the manuscript with assistance of AC. All authors read and approved the final manuscript.
